# AMPK Limits MNNG-Induced Parthanatos by Inhibiting BH3-Only Protein Bim

**DOI:** 10.3390/ijms262110519

**Published:** 2025-10-29

**Authors:** Shuhei Hamano, Tomoe Maruyama, Midori Suzuki, Maki Mitsuya, Takumi Yokosawa, Yusuke Hirata, Atsushi Matsuzawa, Takuya Noguchi

**Affiliations:** 1 Laboratory of Health Chemistry, Graduate School of Pharmaceutical Sciences, Tohoku University, Sendai 980-8578, Japan; 2 Department of Medical Biochemistry, School of Pharmacy, Iwate Medical University, Yahaba 028-3694, Japan

**Keywords:** AMPK, Bim, parthanatos

## Abstract

Parthanatos represents an alternative form of regulated cell death (RCD) mediated by poly (ADP-ribose) polymerase-1 (PARP-1). However, the underlying mechanisms and physiological significance of parthanatos are poorly understood. In this study, we investigated molecular mechanisms of parthanatos in human fibrosarcoma HT1080 cells using biochemical and cellular experiments, and found that parthanatos induced by the alkylating agent *N*-methyl-*N*′-nitro-*N*-nitrosoguanidine (MNNG) is mediated by two alternative pathways that depend on pro-death Bcl-2 family proteins BAX/BAK or Bcl-2-interacting mediator of cell death (Bim). Moreover, we found that MNNG activates AMP-activated protein kinase (AMPK) through PARP-1-dependent ATP depletion, and then AMPK selectively downregulates MNNG-induced parthanatos mediated by Bim but not BAX/BAK. Under unstimulated conditions, expression levels of Bim were below the detection limit. Interestingly, MNNG strongly upregulated the protein expression levels of Bim, but only when the activation of AMPK was inhibited. These observations suggest that the AMPK signaling pathways activated by PARP-1-dependent ATP depletion limit parthanatos by blocking the Bim upregulation triggering Bim-mediated parthanatos. Thus, our results demonstrate a novel relationship between AMPK and parthanatos, which may provide insights into the physiological roles of parthanatos.

## 1. Introduction

Regulated cell death (RCD) is an evolutionarily conserved mechanism that contributes to tissue formation and elimination of damaged cells [1,2]. To date, several forms of RCD, including apoptosis, necroptosis, pyroptosis, ferroptosis, and parthanatos, have been identified, and their distinct mechanisms and functions have been elucidated [3,4]. Poly (ADP-ribose) polymerase-1 (PARP-1) is a nuclear enzyme that mediates poly-ADP-ribosylation (PARylation) of proteins. PARP-1 mainly acts as a sensor of DNA damage, and contributes to DNA repair and genomic stability [5]. On the other hand, recent evidence has suggested that PARP-1 is involved in inflammatory responses that upregulate a series of pro-inflammatory cytokines and nitric oxide (NO) [6,7,8,9,10]. Importantly, severe genotoxic stress initiates the hyperactivation of PARP-1, which alters the function of PARP-1. Hyperactivated PARP-1 induces non-apoptotic regulated cell death called parthanatos by promoting the nuclear translocation of the mitochondrial-associated apoptosis-inducing factor (AIF) that causes large-scale DNA fragmentation and chromatin condensation [11,12]. In particular, it has been reported that several cytotoxic agents, including *N*-methyl-*N*′-nitro-*N*-nitrosoguanidine (MNNG), preferentially induce parthanatos rather than apoptosis [13,14,15]. Therefore, MNNG is widely used to analyze the mechanism of parthanatos induction. MNNG induces DNA damage through alkylation of DNA lesions, leading to hyperactivation of PARP-1 and subsequent cell death [16]. Furthermore, MNNG-induced PARP-1 hyperactivation causes mitochondrial pore formation mediated by pro-apoptotic protein Bcl-2 family members, including BAX and BAK, which allows for the release of AIF from mitochondria into the cytosol [17,18].

AMP-activated protein kinase (AMPK), a multi-subunit protein kinase complex composed of AMPKα (a catalytic subunit), AMPKβ, and AMPKγ (regulatory subunits), is an evolutionarily conserved serine/threonine protein kinase that acts as a master regulator of cellular homeostasis [19]. AMPK is activated by various stresses, and then stimulates the activation of signaling pathways that replenish cellular ATP supplies. In particular, AMPK promotes fatty acid oxidation and autophagy by targeting acetyl-CoA carboxylase 1 (ACC1) and regulatory-associated protein of mTOR (Raptor), respectively [20,21]. On the other hand, several lines of evidence suggest that AMPK is involved in the regulation of RCD as both a pro- and anti-death protein [22,23,24,25,26,27]. Consistent with these reports, AMPK is known to have dual roles, contributing to both tumor suppression and tumor promotion depending on the situation and cell type, although STK11 (LKB1), an upstream factor of AMPK, functions as a tumor suppressor [28,29,30,31,32].

Bcl-2 (B-cell lymphoma-2) family proteins are key regulators of RCD, and are characterized by the presence of four conserved Bcl-2 homology domains (BH domains 1–4) [33]. Bcl-2 family proteins are classified into three subfamilies based on their properties: (1) anti-apoptotic proteins; (2) pro-apoptotic BH3-only proteins; (3) pro-apoptotic pore-forming proteins [34]. The anti-apoptotic proteins and BH3-only proteins competitively regulate the activity of the pore-forming proteins. Activated pore-forming proteins multimerize, forming pores in the outer mitochondrial membrane, inducing mitochondrial outer membrane permeability (MOMP), which induces release of pro-death proteins, including AIF. On the other hand, overexpression of the anti-apoptotic protein Bcl-2 frequently suppresses induction of MOMP in cancer cells, which has been suggested to contribute to resistance to chemotherapy [35,36]. Fibrosarcoma is a malignant tumor that develops in connective tissues such as tendons, ligaments, and the inside of bones. There are two known types of fibrosarcoma: infantile fibrosarcoma and adult fibrosarcoma [37]. Adult fibrosarcoma is generally more malignant than infantile fibrosarcoma [37]. While surgical resection is the primary treatment, chemotherapy is also used as an adjunct to surgery or radiation therapy, but its usefulness is unclear [38]. For this reason, the development of effective chemotherapy is one of the challenges in treating fibrosarcoma.

In this study, we found that MNNG-induced parthanatos is mediated by two pathways: the already-known BAX/BAK-dependent pathway and the newly identified Bim-dependent pathway. Interestingly, AMPK, activated by MNNG-dependent rapid ATP depletion, suppressed MNNG-induced parthanatos by selectively inhibiting the Bim-dependent pathway. Thus, our results suggest that AMPK is an anti-parthanatos factor, suppressing Bim-dependent MOMP and subsequent parthanatos.

## 2. Results

### 2.1. MNNG Induces Parthanatos in a BAX/BAK-Dependent Manner

**Figure 1:**
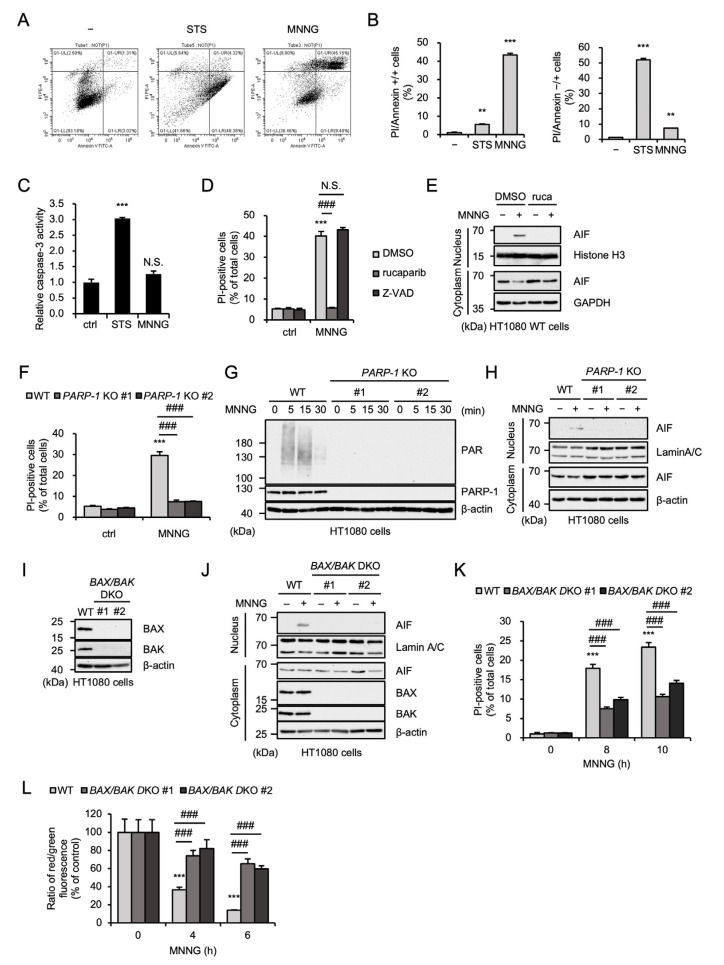
MNNG induces parthanatos in a BAX/BAK-dependent manner. (**A**) HT1080 cells were treated with STS (200 ng/mL for 24 h) or MNNG (30 μM for 6 h) and then cells were labeled with annexin V-FITC and PI. (**B**) Quantitative graphs for PI/annexin-double-positive cells and annexin-positive cells in (**A**). Data shown are the mean ± SEM (*n* = 3). *** *p* < 0.001, ** *p* < 0.01 [vs. MNNG-unstimulated cells]. (**C**) HT1080 cells were treated with STS (200 ng/mL for 8 h) or MNNG (30 μM for 6 h). Caspase-3 activity was measured by colorimetric caspase-3 assay. Data are shown as the ratio of caspase-3 activity versus the control cells (ctrl). Data shown are the mean ± SEM (*n* = 3). *** *p* < 0.001, N.S.: not significant. (**D**) HT1080 cells were treated with 1 μM rucaparib or 20 μM Z-VAD-fmk (Z-VAD) for 60 min and then stimulated with 30 μM MNNG for 8 h. Cell death rate was defined as the percentage of cells with higher PI fluorescence values than when unstimulated. Data shown are the mean ± SEM (*n* = 3). Statistical significance was evaluated by one-way ANOVA, followed by the Tukey–Kramer test; *** *p* < 0.001 [vs. MNNG-unstimulated cells (ctrl)], ### *p* < 0.001, N.S.: not significant [vs. MNNG-stimulated cells (MNNG)]. (**E**) HT1080 cells were treated with 1 μM rucaparib (ruca) for 1 h and then stimulated with 30 μM MNNG for 8 h. The nuclear and cytoplasm lysates were analyzed by immunoblotting with the indicated antibodies. (**F**) WT and *PARP-1* KO HT1080 cells were stimulated with 30 μM MNNG for 8 h. Cell death rate was defined as the percentage of cells with higher PI fluorescence values than when unstimulated. Data shown are the mean ± SEM (*n* = 3). *** *p* < 0.001 [vs. MNNG-unstimulated cells (ctrl)], ### *p* < 0.001 (vs. MNNG-stimulated WT cells). (**G**) WT and *PARP-1* KO HT1080 cells were stimulated with 30 μM MNNG for the indicated periods. Cell lysates were analyzed by immunoblotting with the indicated antibodies. (**H**) WT and *PARP-1* KO HT1080 cells were stimulated with 30 μM MNNG for 8 h. The nuclear and cytoplasm lysates were analyzed by immunoblotting with the indicated antibodies. (**I**) WT and BAX/BAK DKO HT1080 cell lysates were analyzed by immunoblotting with the indicated antibodies. (**J**) WT and *BAX/BAK* DKO HT1080 cells were stimulated with 30 μM MNNG for 8 h. The nuclear and cytoplasm lysates were analyzed by immunoblotting with the indicated antibodies. (**K**) WT and *BAX/BAK* DKO HT1080 cells were stimulated with 30 μM MNNG for the indicated periods. Cell death rate was defined as the percentage of cells with higher PI fluorescence values than when unstimulated. Data shown are the mean ± SEM (*n* = 3). *** *p* < 0.001 [vs. MNNG-unstimulated cells (0 h)], ### *p* < 0.001 (vs. MNNG-stimulated WT cells). (**L**) WT and *BAX/BAK* DKO HT1080 cells were stimulated with 30 μM MNNG for the indicated periods. Mitochondrial membrane potential was measured using a JC-1 probe. Data shown are the mean ± SEM (*n* = 5). *** *p* < 0.001 [vs. MNNG-unstimulated cells (0 h)], ### *p* < 0.001 (vs. MNNG-stimulated WT cells). All data are representative of at least three independent experiments.

Accumulating evidence has demonstrated that MNNG preferentially causes parthanatos, whereas other alkylating agents such as temozolomide mainly induce apoptosis [14,39,40]. In human fibrosarcoma HT1080 cells, propidium iodide (PI) staining showed that cell death induced by staurosporine, a well-known apoptosis inducer, was characterized by PI-negative/annexin-positive cell death, a hallmark of apoptosis, whereas MNNG-induced cell death was characterized by PI/annexin-double-positive cell death (Figure 1A,B). In addition, MNNG failed to stimulate the caspase-3 activation, another hallmark of apoptosis (Figure 1C). Therefore, these observations indicate that MNNG induces non-apoptotic cell death. On the other hand, the PARP-1 inhibitor rucaparib, but not the pan-caspase inhibitor Z-VAD-fmk, inhibits MNNG-induced cell death (Figure 1D). Rucaparib also inhibited the translocation to the nucleus of apoptosis-inducing factor (AIF) triggering parthanatos (Figure 1E) [12]. To confirm the requirement of PARP-1 in MNNG-induced cell death, we investigated *PARP-1*-knockout (KO) HT1080 cells, established in a previous study [41]. As shown in Figure 1F, *PARP-1* KO HT1080 cells showed significant resistance to MNNG-induced cell death, as we expected. In addition, MNNG causes PARP1-dependent poly-ADP-ribosylation (PARylation) and promotes PARP1-dependent AIF nuclear translocation, showing that MNNG stimulates PARP-1 activation and subsequent parthanatos (Figure 1G,H). Pro-apoptotic protein Bcl-2 family members, such as BAX and BAK, contribute to the induction of apoptosis by mediating mitochondrial outer membrane permeabilization (MOMP) that allows release of pro-apoptotic factors, such as cytochrome c and second mitochondria-derived activator of caspases (Smac), from mitochondria [42,43]. Notably, a previous report has demonstrated that BAX is required for MNNG-induced parthanatos in mouse embryonic fibroblasts [17]. Consistent with this observation, MNNG-induced AIF nuclear translocation and subsequent cell death were strongly inhibited in *BAX* and *BAK* double-knockout (*BAX/BAK* DKO) HT1080 cells (Figure 1I–K). Moreover, a JC-1 assay to detect loss of mitochondrial membrane potential (MMP) revealed that MNNG induces MOMP, which is inhibited by *BAX/BAK* double knockout (Figure 1L). Thus, these observations indicate that MNNG induces parthanatos through BAX/BAK-dependent MOMP.

### 2.2. AMPK Negatively Regulates MNNG-Induced Parthanatos

**Figure 2:**
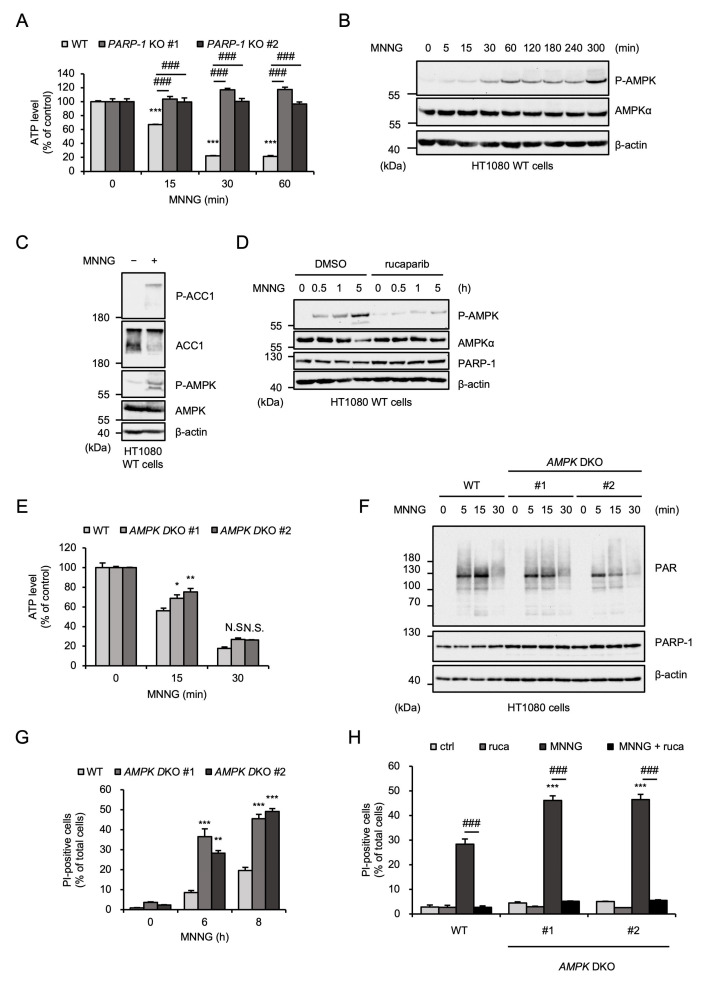
AMPK negatively regulates MNNG-induced parthanatos. (**A**) WT and *PARP-1* KO HT1080 cells were stimulated with 30 μM MNNG for the indicated times. Intracellular ATP level was measured by ATP assay. Data shown are the mean ± SEM (*n* = 3). *** *p* < 0.001 [vs. MNNG-unstimulated cells (0 h)], ### *p* < 0.001 (vs. MNNG-stimulated WT cells). (**B**) HT1080 cells were stimulated with 30 μM MNNG for the indicated periods. Cell lysates were analyzed by immunoblotting with the indicated antibodies. (**C**) HT1080 cells were treated with MNNG (30 μM) for 3 h. Cell lysates were analyzed by immunoblotting with the indicated antibodies. (**D**) HT1080 cells were treated with 1 μM rucaparib for 1 h and then stimulated with 30 μM MNNG for the indicated periods. Cell lysates were analyzed by immunoblotting with the indicated antibodies. (**E**) WT and *AMPK* DKO HT1080 cells were stimulated with 30 μM MNNG for the indicated times. Intracellular ATP level was measured by ATP assay. Data shown are the mean ± SEM (*n* = 3). ** *p* < 0.01, * *p* < 0.05, N.S.: not significant (vs. MNNG-stimulated WT cells). (**F**) WT and *AMPK* DKO HT1080 cells were stimulated with 30 μM MNNG for the indicated periods. Cell lysates were analyzed by immunoblotting with the indicated antibodies. (**G**) WT and *AMPK* DKO HT1080 cells were stimulated with 30 μM MNNG for the indicated periods. Cell death rate was defined as the percentage of cells with higher PI fluorescence values than when unstimulated. Data shown are the mean ± SEM (*n* = 3). *** *p* < 0.001, ** *p* < 0.01 (vs. MNNG-stimulated WT cells). (**H**) WT and *AMPK* DKO HT1080 cells were treated with 1 μM rucaparib (ruca) for 1 h and then stimulated with 30 μM MNNG for 8 h. Cell death rate was defined as the percentage of cells with higher PI fluorescence values than when unstimulated. Data shown are the mean ± SEM (*n* = 3). *** *p* < 0.001 (vs. WT), ### *p* < 0.001 (vs. MNNG-stimulated cells). All data are representative of at least three independent experiments.

Several lines of evidence have demonstrated that MNNG causes PARP-1-mediated ATP depletion during parthanatos [13,44,45]. Indeed, MNNG rapidly and subsequently reduced ATP levels in HT1080 cells (Figure 2A). The rapid depletion of ATP induced by MNNG was not observed in *PARP-1* KO HT1080 cells, indicating that MNNG-induced ATP depletion was mediated by PARP-1 activation (Figure 2A). It is well known that a reduced ATP/ADP ratio is recognized by AMPK and stimulates its activation [19]. As shown in Figure 2B, MNNG clearly stimulated AMPK activation. Consistent with this observation, MNNG promoted phosphorylation of acetyl-CoA carboxylase 1 (ACC1), a typical substrate of AMPK (Figure 2C). Moreover, MNNG-induced AMPK activation was inhibited by the PARP-1 inhibitor rucaparib, which is correlated well with MNNG-induced ATP depletion (Figure 2D). Therefore, MNNG activates AMPK through PARP-1 dependent ATP depletion. We therefore exploited *AMPKα1* and *α2* double-knockout HT1080 cells (*AMPK* DKO HT1080 cells), which were established and characterized in our previous study, to explore the potential roles of AMPK in MNNG-induced parthanatos [25]. At first, MNNG-induced ATP depletion was not affected by the knockout of *AMPK* (Figure 2E). Moreover, the degree of poly-ADP-ribosylation (PARylation) reflecting PARP-1 activation was not altered in *AMPK* DKO HT1080 cells (Figure 2F). These observations support the idea that AMPK is activated by the ATP depletion downstream of the PARP-1 activation. We next investigated the involvement of AMPK in MNNG-induced parthanatos. Interestingly, PI staining revealed that *AMPK* DKO HT1080 cells are highly sensitive to MNNG-induced cell death, and their sensitivity was completely canceled by treatment with rucaparib (Figure 2G,H). Thus, these results indicate that AMPK negatively regulates MNNG-induced parthanatos.

### 2.3. AMPK Limits MNNG-Induced Parthanatos Mediated by BAX/BAK-Independent Pathway

**Figure 3:**
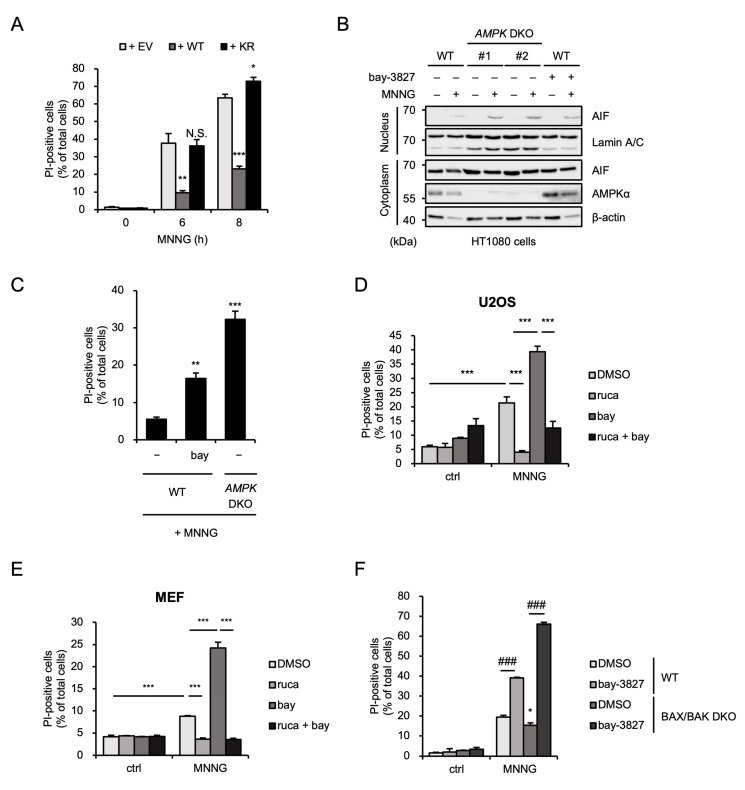
AMPK limits MNNG-induced parthanatos mediated by a BAX/BAK-independent pathway. (**A**) AMPKα1-reconstituted HT1080 cells were stimulated with 30 μM MNNG for the indicated periods. Cell death rate was defined as the percentage of cells with higher PI fluorescence values than when unstimulated. Data shown are the mean ± SEM (*n* = 3). *** *p* < 0.001, ** *p* < 0.01, * *p* < 0.05, N.S.: not significant [vs. MNNG-stimulated empty vector-reconstituted cells (+ EV)]. (**B**) WT and *AMPK* DKO HT1080 cells were treated with 1 μM bay-3827 for 1 h and then stimulated with 30 μM MNNG for 8 h. The nuclear and cytoplasm lysates were analyzed by immunoblotting with the indicated antibodies. (**C**) WT and *AMPK* DKO HT1080 cells were treated with 1 μM bay-3827 for 1 h and then stimulated with 30 μM MNNG for 6 h. Cell death rate was defined as the percentage of cells with higher PI fluorescence values than when unstimulated. Data shown are the mean ± SEM (*n* = 3). *** *p* < 0.001, ** *p* < 0.01 (vs. MNNG-stimulated WT cells). (**D**) U2OS cells were treated with 1 μM rucaparib (ruca) or 1 μM bay-3827 (bay) for 1 h and then stimulated with 30 μM MNNG for 8 h. Cell death rate was defined as the percentage of cells with higher PI fluorescence values than when unstimulated. Data shown are the mean ± SEM (*n* = 3). *** *p* < 0.001. (**E**) MEF cells were treated with 1 μM rucaparib (ruca) or 1 μM bay-3827 (bay) for 60 min and then stimulated with 500 μM MNNG for 20 min. Cell death rates were measured by PI staining 10 h after replacement with fresh medium. Cell death rate was defined as the percentage of cells with higher PI fluorescence values than when unstimulated. Data show the mean ± SEM (*n* = 3). *** *p* < 0.001. (**F**) WT and *BAX/BAK* DKO HT1080 cells were treated with 1 μM bay-3827 for 1 h and then stimulated with 30 μM MNNG for 8 h. Cell death rate was defined as the percentage of cells with higher PI fluorescence values than when unstimulated. Data shown are the mean ± SEM (*n* = 3). * *p* < 0.05 (vs. MNNG-stimulated WT cells), ### *p* < 0.001 (vs. MNNG-stimulated each cell). All data are representative of at least three independent experiments.

We next exploited AMPKα1-reconstituted HT1080 cells, established previously, in order to examine whether the kinase activity of AMPK is required for MNNG-induced parthanatos [25]. As shown in Figure 3A, the increased sensitivity of *AMPK* DKO HT1080 cells to MNNG-induced parthanatos was suppressed by the reconstitution of AMPKα1 wild-type (WT) but not the kinase-dead mutant (K56R mutant) in which lysine (K) 56 was substituted by arginine (R). In addition, the AMPK kinase inhibitor bay-3827 accelerated AIF nuclear translocation and cell death to a similar extent as *AMPK* DKO HT1080 cells (Figure 3B,C). Observations that MNNG-induced cell death was accelerated by bay-3827 and contrarily inhibited by rucaparib were observed in both human osteosarcoma U2OS cells and mouse embryonic fibroblasts (MEFs) (Figure 3D,E). These results therefore suggest that the inhibitory effect of AMPK on MNNG-induced parthanatos is exerted by its kinase activity. We next investigated the effect of bay-3827 on MNNG-induced parthanatos in *BAX/BAK* DKO HT1080 cells. Surprisingly, bay-3827 enhanced MNNG-induced parthanatos even in the absence of BAX and BAK (Figure 3F). Thus, these results suggest that MNNG can initiate parthanatos both in BAX/BAK-dependent and -independent manners, and that AMPK limits MNNG-induced parthanatos mediated by a BAX/BAK-independent pathway.

### 2.4. AMPK Limits MNNG-Induced Upregulation of Pro-Apoptotic Protein Bim

**Figure 4:**
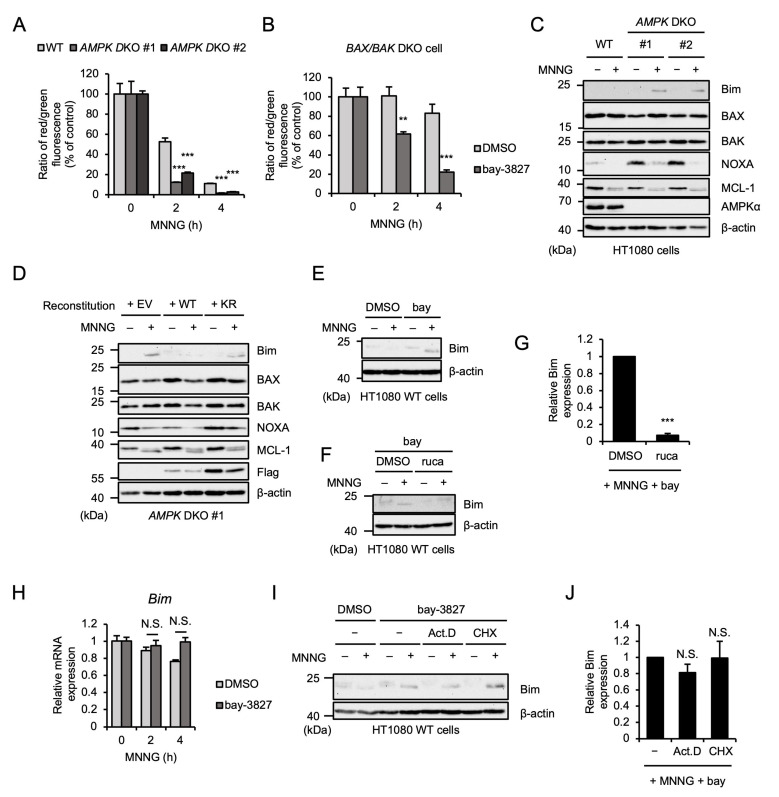
AMPK limits MNNG-induced upregulation of pro-apoptotic protein Bim. (**A**) WT and *AMPK* DKO HT1080 cells were stimulated with 30 μM MNNG for the indicated periods. Mitochondrial membrane potential was measured using a JC-1 probe. Data shown are the mean ± SEM (*n* = 5). *** *p* < 0.001 (vs. MNNG-stimulaed WT cells). (**B**) *BAX/BAK* DKO HT1080 cells were treated with 1 μM bay-3827 for 1 h and then stimulated with 30 μM MNNG for the indicated periods. Mitochondrial membrane potential was measured using a JC-1 probe. Data shown are the mean ± SEM (*n* = 5). *** *p* < 0.001, ** *p* < 0.01 [vs. MNNG-stimulated cells (DMSO)]. (**C**) WT and *AMPK* DKO HT1080 cells were stimulated with 30 μM MNNG for 6 h. Cell lysates were analyzed by immunoblotting with the indicated antibodies. (**D**) AMPKα1-reconstituted HT1080 cells were stimulated with 30 μM MNNG for 6 h. Cell lysates were analyzed by immunoblotting with the indicated antibodies. (**E**) HT1080 cells were treated with 1 μM bay-3827 (bay) for 1 h and then stimulated with 30 μM MNNG for 6 h. Cell lysates were analyzed by immunoblotting with the indicated antibodies. (**F**) HT1080 cells were treated with 1 μM bay-3827 (bay) and 1 μM rucaparib (ruca) for 1 h and then stimulated with 30 μM MNNG for 6 h. Cell lysates were analyzed by immunoblotting with the indicated antibodies. (**G**) The band intensity of Bim normalized with that of β-actin in (**F**). Data are shown as mean ± SEM (*n* = 3). *** *p* < 0.001 [vs. MNNG-stimulated cells]. (**H**) HT1080 cells were treated with 1 μM bay-3827 for 1 h and then stimulated with 30 μM MNNG for the indicated periods. *Bim* mRNA levels were evaluated by quantitative real-time PCR. Data shown are the mean ± SEM (*n* = 3); N.S.: not significant [vs. MNNG-stimulated cells (DMSO)]. (**I**) HT1080 cells were treated with 1 μM bay-3827, 1 μg/mL Actinomycin D (Act. D), and 5 μg/mL cycloheximide (CHX) for 1 h and then stimulated with 30 μM MNNG for 6 h. Cell lysates were analyzed by immunoblotting with the indicated antibodies. (**J**) The band intensity of Bim normalized with that of β-actin in (**I**). Data are shown as mean ± SEM (*n* = 3); N.S.: not significant [vs. MNNG-stimulated cells]. All data are representative of at least three independent experiments.

We next investigated how AMPK limits MNNG-induced parthanatos. At first, we found that MOMP induced by MNNG was enhanced in *AMPK* DKO HT1080 cells (Figure 4A). Moreover, the AMPK kinase inhibitor bay-3827 enhanced MNNG-induced MOMP even in the absence of BAX and BAK (Figure 4B). We therefore speculated that AMPK inactivates pro-apoptotic protein Bcl-2 family members that mediate MNNG-induced MOMP other than BAX and BAK, and thereby limits MNNG-induced parthanatos. We next tested the expression levels of the pro-apoptotic protein Bcl-2 family members that initiate MOMP in HT1080 cells. Interestingly, MNNG increased the expression levels of Bim in *AMPK* DKO HT1080 cells, whereas MNNG failed to do so in WT HT1080 cells (Figure 4C). The reconstitution of AMPKα1 WT but not the KR mutant into *AMPK* DKO HT1080 cells clearly limited the Bim upregulation (Figure 4D). In contrast, the treatment with bay-3827 promoted the upregulation of Bim, which was suppressed by co-treatment with rucaparib (Figure 4E–G). On the other hand, there were no differences between WT and *AMPK* DKO HT1080 cells in the expression levels of BAX, BAK, and myeloid cell leukemia sequence 1 (MCL-1) (Figure 4C). In addition, the expression levels of NOXA were increased in *AMPK* DKO HT1080 cells, whereas the difference disappeared when MNNG was treated (Figure 4C). Collectively, these observations suggest that at least the expression levels of Bim were mediated by PARP-1-dependent AMPK activation. We next examined the mechanisms by which AMPK limits Bim expression. Treatment with bay-3827 did not change the Bim expression at the mRNA level (Figure 4H). Moreover, both the transcriptional inhibitor actinomycin D and the protein synthesis inhibitor cycloheximide failed to suppress the Bim upregulation induced by bay-3827 (Figure 4I,J). AMPK therefore appears to regulate Bim expression at protein levels, but not transcriptional and translational levels.

### 2.5. Bim Is Responsible for the Enhancement of MNNG-Induced Parthanatos in AMPK DKO HT1080 Cells

**Figure 5:**
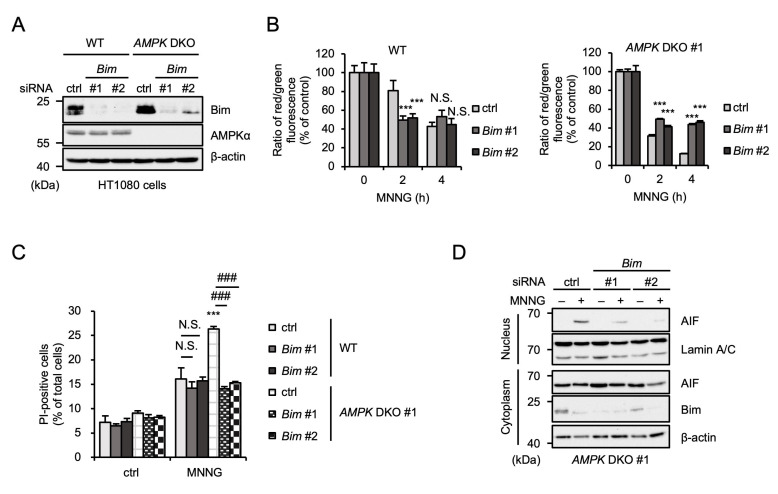
Bim is responsible for the enhancement of MNNG-induced parthanatos in *AMPK* DKO HT1080 cells. (**A**) WT and *AMPK* DKO HT1080 cells were transfected with the indicated siRNA for 48 h. Cell lysates were analyzed by immunoblotting with the indicated antibodies. (**B**) WT and *AMPK* DKO HT1080 cells were transfected with the indicated siRNA and then stimulated with 30 μM MNNG for the indicated periods. Mitochondrial membrane potential was measured using a JC-1 probe. Data shown are the mean ± SEM (*n* = 5). *** *p* < 0.001, N.S.: not significant [vs. MNNG-stimulated cells (ctrl)]. (**C**) WT and *AMPK* DKO HT1080 cells were transfected with the indicated siRNA for 48 h and then stimulated with 30 μM MNNG for 6 h. Cell death rate was defined as the percentage of cells with higher PI fluorescence values than when unstimulated. Data shown are the mean ± SEM (*n* = 3). *** *p* < 0.001 [vs. MNNG-stimulated WT cells (ctrl)], ### *p* < 0.001, N.S.: not significant [vs. MNNG-stimulated each cell (ctrl)]. (**D**) *AMPK* DKO HT1080 cells were transfected with the indicated siRNA for 48 h and then stimulated with 30 μM MNNG for 6 h. The nuclear and cytoplasm lysates were analyzed by immunoblotting with the indicated antibodies. All data are representative of at least three independent experiments.

We next performed knockdown experiments to examine whether Bim is involved in MNNG-induced parthanatos (Figure 5A). Interestingly, knockdown of *Bim* did not affect MNNG-induced MOMP in WT HT1080 cells, whereas MNNG-induced MOMP was suppressed by the *Bim* knockdown in *AMPK* DKO HT1080 cells (Figure 5B). Consistent with these observations, the *Bim* knockdown suppressed MNNG-induced parthanatos only in *AMPK* DKO HT1080 cells (Figure 5C). Moreover, AIF nuclear translocation was clearly suppressed by *Bim* knockdown (Figure 5D). Therefore, these results suggest that Bim is responsible for the enhancement of MNNG-induced MOMP and subsequent parthanatos in *AMPK* DKO HT1080 cells.

### 2.6. Bim Mediates MNNG-Induced Parthanatos Independently of BAX/BAK

**Figure 6:**
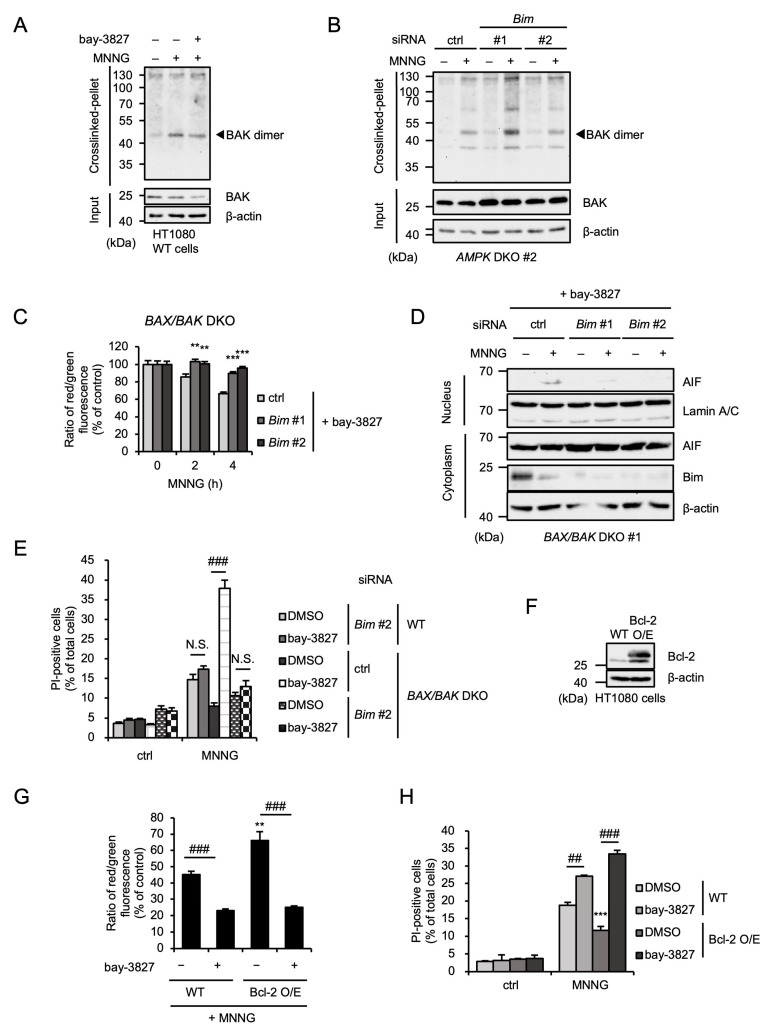
Bim mediates MNNG-induced parthanatos independently of BAX/BAK. (**A**) HT1080 cells were treated with 1 μM bay-3827 for 1 h and then stimulated with 30 μM MNNG for 6 h. Crosslinked-pellets and Input were analyzed by immunoblotting with the indicated antibodies. (**B**) *AMPK* DKO HT1080 cells were transfected with the indicated siRNA for 48 h and then stimulated with 30 μM MNNG for 6 h. Crosslinked-pellets and Input were analyzed by immunoblotting with the indicated antibodies. (**C**) *BAX/BAK* DKO HT1080 cells were transfected with the indicated siRNA. After 48 h, cells were treated with 1 μM bay-3827 for 1 h and then stimulated with 30 μM MNNG for the indicated periods. Mitochondrial membrane potential was measured using a JC-1 probe. Data shown are the mean ± SEM (*n* = 5). *** *p* < 0.001, ** *p* < 0.01 [vs. MNNG-stimulated cells (ctrl)]. (**D**) *BAX/BAK* DKO HT1080 cells were transfected with the indicated siRNA. After 48 h, cells were treated with 1 μM bay-3827 for 1 h and then stimulated with 30 μM MNNG for 6 h. The nuclear and cytoplasm lysates were analyzed by immunoblotting with the indicated antibodies. (**E**) WT and *BAX/BAK* DKO HT1080 cells were transfected with the indicated siRNA. After 48 h, cells were treated with 1 μM bay-3827 for 1 h and then stimulated with 30 μM MNNG for 6 h. Cell death rate was defined as the percentage of cells with higher PI fluorescence values than when unstimulated. Data shown are the mean ± SEM (*n* = 3). ### *p* < 0.001, N.S.: not significant [vs. MNNG-stimulated each cell (DMSO)]. (**F**) WT and Bcl-2-overexpressing (Bcl-2 O/E) HT1080 cell lysates were analyzed by immunoblotting with the indicated antibodies. (**G**) WT and Bcl-2-overexpressing (Bcl-2 O/E) HT1080 cells were treated with 1 μM bay-3827 for 1 h and then stimulated with 30 μM MNNG for 4 h. Mitochondrial membrane potential was measured using a JC-1 probe. Data shown are the mean ± SEM (*n* = 5). ** *p* < 0.01 (vs. MNNG-stimulated WT cells), ### *p* < 0.001, N.S.: not significant (vs. each MNNG-stimulated cell). (**H**) WT and Bcl-2-overexpressing (Bcl-2 O/E) HT1080 cells were treated with 1 μM bay-3827 for 1 h and then stimulated with 30 μM MNNG for 6 h. Cell death rate was defined as the percentage of cells with higher PI fluorescence values than when unstimulated. Data shown are the mean ± SEM (*n* = 3). *** *p* < 0.001 (vs. MNNG-stimulated WT cells), ### *p* < 0.001, ## *p* < 0.01 [vs. each MNNG-stimulated cell (DMSO)]. All data are representative of at least three independent experiments.

Given that AMPK limits MNNG-induced parthanatos mediated by the BAX/BAK-independent pathway, the effect of Bim on MNNG-induced parthanatos may be exerted regardless of the expression of BAX and BAK. At first, we evaluated the dimerization of BAK, an index of its activation [46]. As shown in Figure 6A,B, the dimerization assay revealed that MNNG promotes BAK dimerization, which was not affected by treatment with bay-3827 or *Bim* knockdown. Therefore, both AMPK and Bim do not appear to affect MNNG-induced BAK activation. On the other hand, enhancement of MNNG-induced MOMP induced by bay-3827 in *BAX/BAK* DKO HT1080 cells, shown in Figure 4B, was clearly canceled by *Bim* knockdown (Figure 6C). Consistent with this observation, MNNG-induced AIF nuclear translocation and subsequent parthanatos in the presence of bay-3827 were also canceled by *Bim* knockdown (Figure 6D,E). To confirm the redundancy between BAX/BAK and Bim, we established HT1080 cells overexpressing Bcl-2, a suppressor of BAX/BAK, as BAX/BAK-dependent MOMP-deficient cells (Figure 6F). As shown in Figure 6E, MNNG-induced MOMP was significantly inhibited in Bcl-2-overexpressing HT1080 cells. However, the treatment with bay-3827 promoted MNNG-induced MOMP in Bcl-2-overexpressing HT1080 cells, to a similar extent as in WT HT1080 cells (Figure 6G). Moreover, MNNG-induced parthanatos was also inhibited in Bcl-2-overexpressing HT1080 cells, but bay-3827 reversed this inhibition (Figure 6H). Taken together, our results suggest that MNNG-induced parthanatos is mediated by both BAX/BAK- and Bim-dependent pathways, and AMPK negatively regulates MNNG-induced parthanatos through the selective inhibition of Bim-dependent pathways.

## 3. Discussion

**Figure 7:**
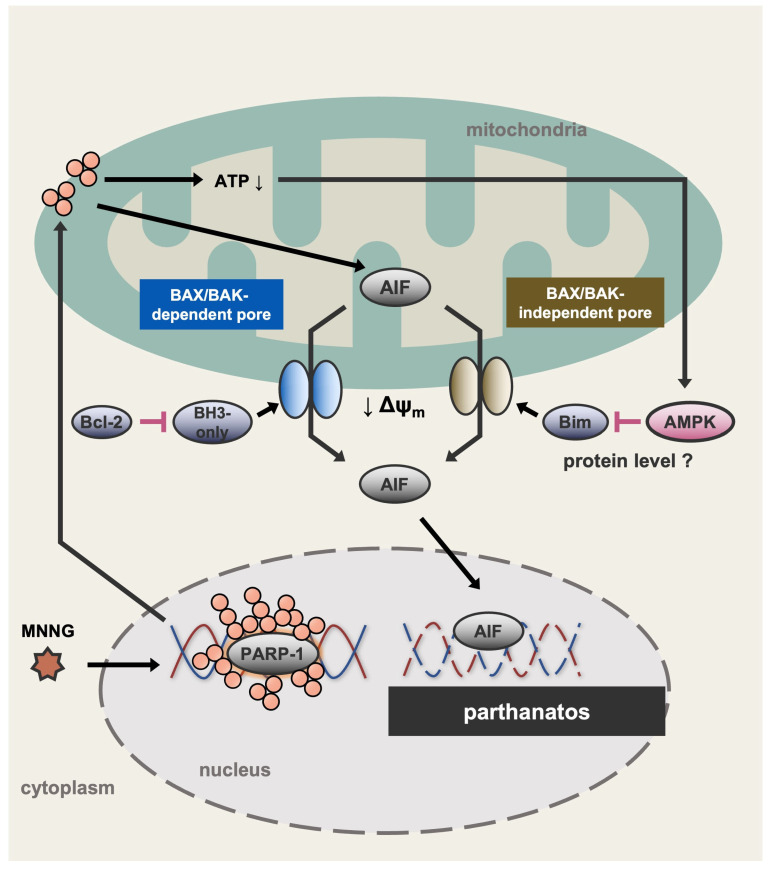
A schematic model to explain our study. MNNG induces parthanatos through both BAX/BAK- and Bim-dependent pathways. Meanwhile, MNNG causes PARP-1-dependent rapid ATP depletion, leading to the activation of AMPK. Interestingly, activated AMPK suppresses the upregulation of Bim induced by MNNG, possibly at the protein level, and thereby limits Bim-dependent parthanatos. In the absence of the AMPK activation, upregulated Bim individually promotes MNNG-induced MOMP, and therefore induces parthanatos independently of BAX and BAK. ATP↓: ATP depletion. ↓ Δψm: Decrease in mitochondrial membrane potential.

In this study, we demonstrated that MNNG induces parthanatos in HT1080 cells through BAX/BAK activation. Furthermore, AMPK, activated by PARP-1-dependent rapid ATP depletion, limits MNNG-induced parthanatos. Further analyses revealed that AMPK limits MNNG-induced parthanatos by suppressing the upregulation of Bim. Interestingly, Bim promoted MNNG-induced parthanatos even in the absence of BAX and BAK, suggesting that Bim induces MOMP independently of BAX and BAK (Figure 7). Analysis of the mechanism of AMPK-mediated suppression of Bim revealed that MNNG-induced upregulation of Bim is transcription- and translation-independent, suggesting that Bim is somehow regulated at the protein level. It has been reported that phosphorylation of Bim promotes its poly-ubiquitination and subsequent proteasomal degradation [47,48]. Therefore, we speculate that AMPK may directly or indirectly induce Bim phosphorylation, leading to its degradation.

Our results suggest that Bim, whose expression is upregulated by PARP-1 overactivation, contributes to the decrease in mitochondrial membrane potential. Bim, a member of the Bcl-2 family of proteins, is known to promote MOMP induction by suppressing the function of anti-apoptotic factors and by interacting with pore-forming proteins such as BAX and BAK [49]. Interestingly, a recent study has suggested that Bim also induces a decrease in mitochondrial membrane potential in a BAX/BAK-independent manner [50].

Our study supports this finding, although further research is needed to clarify the mechanisms by which Bim promotes MOMP induction independently of BAX and BAK. In this regard, there is the possibility that Bim promotes MOMP induction through Bcl-2-related ovarian killer (BOK), because BOK has recently been reported to have effector functions similar to those of BAX and BAK [51]. Furthermore, the question remains as to why MNNG induces parthanatos rather than apoptosis in the first place. Although further analysis is required to answer this critical question, a previous report may provide a hint to answer this question [52]. The report has demonstrated that apoptosis induction requires a substantial amount of intracellular ATP, which means that apoptosis is less likely to occur under ATP-depleted conditions [52]. Hence, when ATP depletion occurs due to the treatment of MNNG, apoptosis is not induced, and it is possible that parthanatos is induced as an alternative cell death.

AMPK has been reported to have dual roles as an inhibitor and promoter of cancer progression [30,31]. Since cancer cells are generally found in low-energy environments, it is possible that the activation of AMPK, particularly in cancer cells, negatively regulates Bim expression [32]. Indeed, Bim, known as a tumor suppressor, has been reported to be downregulated in many cancer cells [53,54]. Furthermore, in various cancer cells, overexpression of Bcl-2 inhibits BAX/BAK-dependent pore formation, resulting in resistance to anticancer therapies [55,56]. Interestingly, our results demonstrated that the AMPK inhibitor can promote parthanatos in cancer cells. Therefore, combination therapy with the AMPK inhibitor and the alkylating agent that activates PARP-1 may be an effective treatment strategy against cancer. This idea is supported as a strategy for applying parthanatos in anticancer therapies [57,58]. Furthermore, it has been reported that the cell death induced by the anticancer drug streptozotocin, a nitrosourea alkylating agent similar to MNNG, is dependent on PARP-1 [59]. Taken together, elucidating the regulatory mechanisms of Bim-mediated parthanatos, including AMPK-dependent negative regulation, may be a promising approach to develop more effective anticancer pharmaceuticals.

## 4. Materials and Methods

### 4.1. Cell Culture and Reagents

Human fibrosarcoma cell line HT1080 cells were obtained from JCRB Cell Bank (Japanese Collection of Research Bioresources Cell Bank, Osaka, Japan). Human osteosarcoma U2OS cells were obtained from ATCC. HT1080 cells, U2OS cells, and MEFs were cultured in Dulbecco’s Modified Eagle Medium (DMEM) (Nacalai Tesque, Kyoto, Japan), 5% heat-inactivated fetal bovine serum (FBS) (Nichirei Biosciences Inc. Tokyo, Japan), and 1% penicillin–streptomycin solution (Nacalai Tesque) at 37 °C under a 5% CO_2_ atmosphere. All reagents were purchased from commercial sources: Dimethyl sulfoxide (DMSO), Actinomycin D (Act.D) (Wako, Tokyo, Japan), cycloheximide (CHX) (Nacalai Tesque), Z-VAD-fmk (Z-VAD) (Peptide Institute, Osaka, Japan), rucaparib, bay-3827 (Selleck Chemicals, Houston, TX, USA), *N*-methyl-*N*′-nitro-*N*-nitrosoguanidine (MNNG) (Tokyo Chemical Industry, Tokyo, Japan), and staurosporine (STS) (Santa Cruz, Dallas, TX, USA). The antibodies used were against PARP-1 (B-10), Bim (H-5), BAK (AT38E2), BAX (6A7), Bcl-2 (C-2), β-actin (C4), Lamin A/C (636), Histone H3 (1G1), GAPDH (0411), MCL-1 (22), NOXA (F-3) (Santa Cruz, Dallas, TX, USA), P-AMPK (T172) (40H9), AMPKα (F6), AIF (D39D2), Poly ADP-ribose (PAR) (E6F6A), ACC1 (C83B10), P-ACC1 (S79) (Cell Signaling, Danvers, MA, USA), and Flag (Sigma, St. Louis, MO, USA).

### 4.2. Generation of Knockout Cell Lines

All knockout (KO) cells were established as described previously [60]. The targets of guide RNAs (gRNAs) were regions in exon 1 of the *AMPKα1* gene (5′-GAAGCAGAAACACGACGGGC-3′), in exon 4 of the *AMPKα2* gene (5′-GGATTACTGTCATAGGCATA-3′), in exon 1 of the *PARP-1* gene (5′-GAGTCGAGTACGCCAAGAGC-3′), in exon 3 of the *BAX* gene (5′-CGAGTGTCTCAAGCGCATCG-3′), and in exon 2 of the *BAK* gene (5′-GCTGCTAGGTTGCAGAGGTA-3′). Viral vectors were produced and virus-infected cells were selected using the method previously described [61]. To determine the mutations of *AMPKα1*, *AMPKα2*, *PARP-1*, *BAX*, and *BAK*, their genomic sequences were determined by using the following primers: 5′-CGCAGACTCAGTTCCTGGAG-3′ and 5′-CAGCCCTGGAAAGAAGGGAC-3′ for *AMPKα1*; 5′-ATGCAGTTTCTTTTGTGCTTGA-3′ and 5′-CATGGTACAGAACGTACAAGGT-3′ for *AMPKα2*; 5′-GCATCAGCAATCTATCAG-3′ and 5′-CTTCCCGGACACAGTTAA-3′ for *PARP-1*; 5′-CGTTGGCCTGTTGCTTTTCA-3′ and 5′-CACCTTGAGCACCAGTTTGC-3′ for *BAX*; 5′-CCATCAGCAGGAACAGGAGG-3′ and 5′-GTTCTGCCTGAGCTGTCCAT-3′ for *BAK*. Each cloned cell was numbered #1 or #2.

### 4.3. Generation of Stable Cell Line

AMPKα1-reconstituted and Bcl-2-overexpressing (Bcl-2 O/E) HT1080s were established using retrovirus as previously described [62].

### 4.4. siRNA Transfection

HT1080 cells were transfected with siRNAs targeting *Bim* (*Bim* #1; 5′-CACCGUGUCCAUUACAGCAGA-3′, *Bim* #2; 5′-CGGCCUAUUCUCAGAGGAUUA-3′) or a non-targeting siRNA pool (#D-001206-13, Dharmacon, Lafayette, CO, USA) as a control (ctrl) using Lipofectamine RNAiMAX from Invitrogen (Waltham, MA, USA) (#13778150), according to the instructions.

### 4.5. Immunoblot

Isolation of cell extract, SDS-PAGE, blotting, and chemiluminescence were precisely described in our previous report [63].

### 4.6. Nuclear Extraction

Nuclear extraction was conducted as described in our previous report [64].

### 4.7. Mitochondrial Membrane Potential Assay

Mitochondrial membrane potential was evaluated by the JC-1 MitoMP Detection Kit (MT09) (Wako) according to the instructions. Briefly, cells were incubated with 2 µM JC-1 dye at 37 °C. After 1 h, the cells’ fluorescence intensity was measured by SPECTRA max GEMINI XPS (Molecular Devices, San Jose, CA, USA). The ratio of red/green fluorescence was defined as the mitochondrial membrane potential, and quantification was performed with the mitochondrial membrane potential of unstimulated cells set at 100%.

### 4.8. Dimerization Assay

The dimerization assay was conducted as described in our previous report [9].

### 4.9. Propidium Iodide (PI) Staining and PI/Annexin Staining

PI staining using a CytoFLEX (BECKMAN COULTER, Tokyo, Japan) was conducted as described in our previous report [65]. PI/annexin staining was performed as described in our previous report [25]. Briefly, cells were incubated with annexin V-FITC (MBL, Tokyo, Japan) and 2 µg/mL PI (Nacalai Tesque) for 15 min. Fluorescent cells were detected by CytoFLEX, and dead cells were analyzed by using CytExpert (version 2.4) (BECKMAN COULTER).

### 4.10. Quantitative Real-Time PCR (qRT-PCR)

Isolation of total RNA and template cDNA were performed as described previously [66]. The primers used for qRT-PCR were as follows: *Bim*-forward, 5′-AAGAGTTGCGGCGTATTGGA-3′; *Bim*-reverse, 5′-ACCAGGCGGACAATGTAACG-3′; *GAPDH*-forward, 5′-AACAGCCTCAAGATCATCAGC-3′; *GAPDH*-reverse, 5′-GGATGATGTTCTGGAGAGCC-3′.

### 4.11. ATP Assay

The ATP assay was performed by using the Intracellular ATP assay kit ver. 2 (cosmobio, Tokyo, Japan) according to the protocol. Data were normalized to control (100%) without stimulus.

### 4.12. Colorimetric Caspase Assay

The caspase assay was performed as described in our previous report [61]. Briefly, The cells were lysed in the cell lysis buffer included in the Caspase-3 Colorimetric Assay Kit (Biovision, Milpitas, CA, USA). After centrifugation at 15,000 rpm for 15 min, the cell extracts were mixed with caspase reaction buffer [10 mM Tris-HCl (pH 7.4), 150 mM NaCl, 0.1% CHAPS, 2 mM MgCl_2_, 5 mM EGTA, and 1 mM DTT] supplemented with 100 µM DEVD-pNA (Biovision). After incubation at 37 °C for 1 h, the activity of caspase-3 was determined by measuring the absorbance at 405 nm using SpectraMax Paradigm (Molecular Devices).

### 4.13. Statistical Analysis

Statistical analyses using Prism software (version 9.5.1) (GraphPad Software, La Jolla, CA, USA) were performed as described previously [67].

## Data Availability

The data presented in this study are available in article.
